# Shock and awe: Economic sanctions and relative military spending

**DOI:** 10.1177/00223433251331486

**Published:** 2025-05-16

**Authors:** Yuleng Zeng, Andreas Dür

**Affiliations:** Department of International Relations and International Organization, University of Groningen, the Netherlands; Department of Political Science, University of Salzburg, Austria

**Keywords:** Economic sanctions, military spending, trade network

## Abstract

Economic sanctions could cause substantial harm to target states, forcing them to undertake tough guns-versus-butter trade-offs. Although existing research has argued that sanctioned countries reduce their military spending in absolute terms, it is unclear whether they do trade more guns for butter in relative terms. We argue that in the short run, sanctioned states have an incentive to channel proportionally more resources to the military for two primary reasons. First, this allows them to signal their resolve not to back down to sanctioning states and potentially maintain their bargaining leverage. Second, higher relative military spending can strengthen leaders’ hold on power by improving their ability to co-opt and repress political opponents. However, this combined incentive to signal resolve and consolidate power weakens after the initial economic and political shocks. As such, we also expect that the increase in relative military spending will diminish gradually. To test our theory, we propose a new measurement of sanction shocks that carefully accounts for the salience, costs, and duration of different sanction episodes. Using this measure, we apply dynamic panel modeling to examine the military spending of 166 countries from 1962 to 2015. We find strong support for our theoretical expectations. In response to sanction shocks, target states choose to spend proportionally more on the military; this increase peaks in the first few years and dissipates over time. These results hold important implications for research on both economic sanctions and military spending.

In response to Russia’s invasion of Ukraine, Western countries threatened and eventually imposed unprecedented economic sanctions aiming to ‘shock and awe’ Moscow (Alfonsi, 2022). The goal was to force Russia to either abandon the invasion or continue with a heavy toll on its economic health and military capacity. A similar rationale also holds for other prominent cases, such as the United Nations (UN) sanctions against Iraq in 1990 or the renewed United States (USA) sanctions against Iran imposed by the first Trump administration to exert ‘maximum pressure’.

While existing research has shown that such economic sanctions can constrain target states’ military spending in absolute terms (Batchelor et al., 2002; Dizaji and Farzanegan, 2021; Farzanegan, 2022), it remains unclear whether targets really trade guns for butter. Sanctions could compel target governments to divert money from the military to cover spending gaps in other areas and shore up economic growth (Escribà-Folch, 2012; Peksen, 2017). Some sanctions also specifically target the military sector (e.g. arms embargoes or targeted sanctions against military officials), whereas other types of government expenditures are less affected, potentially leading to a relative decline in military budgets.

That said, facing a weakened economy and a smaller budget, embattled leaders may also find it attractive to increase relative military spending, which can more directly affect their political survival (Bueno de Mesquita et al., 2005). Illustratively, after the UN arms embargo in 1963, the South African government chose to increase military spending as a share of GDP from 2.1% in 1963 to 2.7% in the following year and kept it at above 2.6% throughout the rest of the 1960s (SIPRI, 2019). Similarly, Western countries enacted sanctions against Russia in 2014, with Russian military spending as a share of GDP going up from 3.8% in 2013 to 5.4% in 2016 before starting to decline again (SIPRI, 2019). Considering these potentially opposing incentives for governments, we ask: How do sanctions affect target states’ *relative* military spending (i.e. military spending as a share of the economy or the government budget)?

To address this question, we argue that in the initial stage, target countries have an incentive to increase their relative military spending to counter perceived threats from inside and outside the state. With respect to internal threats, increased relative military expenditure in the face of sanctions helps leaders consolidate their power by channeling resources to domestic political supporters and deterring potential challengers. In terms of external threats, increased relative military spending allows target states to signal their determination to sender states and potentially grab some *fait accompli* before their military power and bargaining leverage deteriorate further. As sanctions prolong, the political and economic shocks should weaken over time. As such, the benefits and incentives to boost military spending will dissipate.

To test the foregoing theoretical expectations, we propose a new measure of sanction shocks and apply dynamic panel modeling to examine the military spending of 166 countries from 1962 to 2015. We define sanction shocks as the substantial economic or political disruptions caused by the onset of sanctions that are (expected to be) implemented in a forceful and costly manner. Our measurement accounts for the salience, duration, and costs of different sanction episodes. Using this measure, we find that states encountering sanction shocks tend to allocate a higher portion of their economic resources for military spending. This incentive, however, only exists during the early stage.

The results hold important implications for the sanctions and military spending literature. So far, research in this field has focused chiefly on *absolute* rather than *relative* military spending in the aftermath of sanctions. The finding that states under economic sanctions reduce absolute military spending in the face of stricter budget constraints, however, is not surprising.^1^ By contrast, focusing on relative military spending can better address the guns-versus-butter trade-offs that sanctioned states are forced to make (Powell, 1993). For instance, if sanctions can be deployed to counteract adverse power shifts, we need to understand how states react strategically to the trade-offs (Carter et al., 2021; McCormack and Pascoe, 2015).

Among the few studies analyzing relative military spending, the empirical results are mixed. Escribà-Folch (2012) finds that facing sanctions, personalist regimes cut back military expenditures while military regimes increase them. Dizaji and Farzanegan (2021) find that economic sanctions reduce Iran’s relative military spending, with multilateral sanctions having a much more substantial impact than unilateral ones. By contrast, Farzanegan (2011) finds a consistently positive effect of oil revenue shocks on military expenditure as a share of government expenditure in the case of Iran. Dizaji (2024) finds the effects of oil shocks on Middle Eastern countries in the initial years are positive and will turn negative after three to four years. On top of these mixed results, the studies just mentioned focus on either one country or a particular group of countries. Our study moves the field forward by considering the different kinds of sanction shocks at a global level. We also proffer a novel operationalization of sanction shocks that carefully considers the issue salience, the political and economic costs, and the design of sanctions.

The finding that all else equal, states encountering sanction shocks allocate proportionally more resources to the military in the short run can also help explain why sanctions are not always effective (Bapat et al., 2013; Pape, 1997) and why their destabilizing effects are not so clear-cut (Licht, 2011; Marinov, 2005). Clearly, many target states have the policy tools to counteract foreign pressure and consolidate domestic power during the period that is most critical for sanctions success (Dizaji and van Bergeijk, 2013; Peksen and Drury, 2010). By focusing on target states’ reactions, the article can also shed light on the unintended consequences of sanctions. For example, the humanitarian disaster in Iraq during the 1990s took place against the backdrop of a high proportion of military spending in the early stage of sanctions against the country.^2^ We also see similar stories of civilians in sanctioned countries plummeting into poverty, starvation, and illness while the governments divert limited resources to the military and drain social investment (e.g. Syria, North Korea, and Venezuela). Finally, sanctions’ impact on relative military spending patterns holds broader implications as the latter can affect the probability of coups d’etat (Leon, 2014), democratic transitions (Clardie, 2011), countries’ external debt (Smyth and Kumar Narayan, 2009), and economic growth (d’Agostino et al., 2017; Dunne, 2012).

## Sanctions and military spending

Military spending refers to all current and capital expenditure on military activities and forces. It encompasses spending for military personnel, maintenance of the armed forces, arms purchases, military operations, military research, and military construction (Perlo-Freeman, 2017). Existing studies of military expenditure have identified several determinants at both the international and domestic levels. At the international level, countries tend to spend more on the military if they face external threats (Nordhaus et al., 2012). In particular, military budgets are expanded when states encounter potential challenges from (strategic) rivals (Dunne and Perlo-Freeman, 2003; Fordham and Walker, 2005; Hewitt, 1992; Mintz and Ward, 1989) or are undergoing arms races (Fordham, 2004; Nordhaus et al., 2012). Additionally, international development aid can be abused to purchase weapons (Collier and Hoeffler, 2007). At the domestic level, different political institutions vary in terms of the restraints they impose on military budgets (Acemoglu et al., 2010; Albalate et al., 2012; Goldsmith, 2003; Kim et al., 2013). The quality of government also matters. For instance, corruption can lead to higher military expenditure (Gupta et al., 2001; Thorpe, 2014). Finally, leaders facing a potential economic downturn can use military spending as a welfare policy tool in disguise to spur economic growth (Mintz and Hicks, 1984; Mintz and Stevenson, 1995; Whitten and Williams, 2011).

Economic sanctions are ‘actions that one or more countries take to limit or end their economic relations with a target country in an effort to persuade that country to change its policies’ (Morgan et al., 2014). Given this definition, we recognize that not all policy issues are salient and sanctions are not created equal. Some sanctions are threatened, imposed, and enforced in a much stronger fashion than others, resulting in substantial economic and political disruptions in a target state. We label these as *sanction shocks*. Encountering sanction shocks, target states will face a weaker economy and have less to spend on the military. In this regard, it is unsurprising that some earlier research has found that sanction-shocked countries cut back military expenditure in absolute terms (Batchelor et al., 2002; Dizaji and Farzanegan, 2021).

However, sanction shocks can also heighten target states’ threat perception (Kaempfer and Lowenberg, 1999; Peterson and Drury, 2011; Wood, 2008). Existing studies have demonstrated that as perceived external or internal threats increase, states are more likely to expand their military budgets (Afesorgbor, 2019; Nordhaus et al., 2012). Despite the economic shocks, targets often have the policy option of shifting resources away from other uses, such as education and health, towards the military (Escribà-Folch, 2012). They also have the fiscal option of increasing tax or resorting to borrowing to finance military spending (Carter et al., 2021; Shea, 2016). It is therefore not clear whether sanctions will cause the *share* of spending on the military to increase or decrease.

We argue in the following that in the short run, states that encounter sanction shocks have stronger incentives to increase their relative military spending because it can help both demonstrate their resolve and capacity and consolidate their political power. In short, an increase in relative military spending can help target governments maintain (or even boost) their bargaining leverage against foreign and domestic adversaries. However, these shocks cannot last indefinitely. The sanctioned economy and government will adjust accordingly in the medium to longer terms. As a result, the positive effects of sanction shocks will dissipate over time.

### Signaling resolve and capacity

Sanctions can be understood as a bargaining process between sender and target states. Sender states seek to weaken target states’ bargaining leverage by forcing them to endure economic costs and make harsh guns-versus-butter trade-offs (Carter et al., 2021). In doing so, they have to accept at least some costs driven by the economic disruption. As such, there is always a compromise that both sides would prefer to avoid the inefficient economic attrition (Drezner, 2003). Sanctions are initiated due to states’ uncertainty about an opponent’s determination and capacity. Once this uncertainty is sufficiently reduced, sanctions will likely be terminated (Krustev and Morgan, 2011).

How would the policy choices over relative military spending affect this dynamic bargaining process? We begin by assuming that, on average, sanctioned targets should expect a weaker economy when encountering sanction shocks. In this situation, there are essentially three choices for the target: (a) maintaining (or even increasing) the existing level of military spending, (b) reducing military spending at a slower rate than the decline of the economy, and (c) reducing military spending at a faster rate than the decline of the economy. The first two options will result in an increase in relative military spending, while the last one in a decrease thereof. We argue that option (a) and, to a lesser extent, option (b) are attractive to target leaders because boosting relative military spending can help them signal their resolve and capacity toward sender states, which can potentially help them reach a better deal.

To begin with, investing in the military, even when the economy is in bad shape, can be a signaling tool of resolve. It can demonstrate a leader’s determination to withstand sanctions in a more credible manner since the sunk costs of military expenditures are relatively easy to observe (Fearon, 1997). This is particularly important since the signaling mechanism during economic coercion often fails to work effectively as sender states avoid imposing economic costs on themselves (Dafoe and Kelsey, 2014; Lektzian and Sprecher, 2007; Whang et al., 2013) and target states actively turn to the help of third-party actors (Early, 2015; Peterson, 2020).

Relatedly, sanctioned states might be incentivized to take other costly signaling options (e.g. deploying troops to a disputed territory or escalating the intensity of fighting on the battlefield) to demonstrate their resolve and power (Slantchev, 2011), which further adds to the increase in relative military spending. Admittedly, target states could be bluffing. But they are incentivized to bluff exactly because of the strategic benefits. Regardless of their true intention or capacity, the end outcome is that sanctioned states will find it more attractive to increase relative military spending (i.e. options (a) and (b) mentioned earlier).

For example, after the USA imposed sanctions on North Korea in 2002, the latter signaled multiple times that if the USA did not change its negotiation stance, it would no longer adhere to ‘the self-imposed missile moratorium’ (Arms Control Association, 2020). North Korea’s military spending as a share of GDP increased from 16.5% of GDP in 2002 to 17.7% in 2003, 18.8% in 2004, and 20% in 2006 (US Department of State, 2012). A decade later, facing increased sanctions, North Korea raised its relative military spending from 22% in 2012 to 23.9% in 2013, and 24% in 2014 (US Department of State, 2021). More recently, amid unsuccessful efforts to lift sanctions, North Korea again bumped up the spending from 24.5% in 2018 to 26.4% in 2019 (US Department of State, 2021). In December 2019, Kim Jong-un changed the previous year’s focus on economic development and spoke to the ruling central committee about the need to refocus on the military even at the price of economic growth (Jo, 2020). Pointing to the stalled talks with the USA, he claimed that ‘a leap in developing the ultra-modern national defence science would make our great military and technical power irreversible’ and that North Korea should further harden ‘our resolution never to barter the security and dignity of the state’ (KCNA Watch, 2020). That is, by increasing relative military spending, Kim aimed to demonstrate that the progress of North Korea’s military power is ‘irreversible’ and that he will not budge despite US demands.

### Consolidating power

Aside from the foregoing bargaining and signaling calculations, target leaders often face potentially destabilizing effects of sanctions at the domestic level. Since sanctions are designed to bring direct economic punishment upon targets, target leaders have fewer resources to buy off supporters (Kaempfer and Lowenberg, 1988; McGillivray and Stam, 2004). The reduced resources, combined with the signaling effect of international opposition, can trigger protests and threaten an incumbent’s hold on power (Allen, 2008a; Grauvogel et al., 2017; Marinov, 2005; cf. Licht, 2017).

Expecting such effects, target leaders can counteract by increasing relative military spending. Investing in the military improves and demonstrates their ability to step up repression if necessary in the future (Escribà-Folch and Wright, 2010). Reallocating resources from low-priority spending to the military can also help leaders channel resources to their supporters and co-opt political opponents (Cortright et al., 2000; McLean and Whang, 2021; Rowe, 2001; Woodward, 1995). Aside from repression and co-optation, leaders can utilize foreign policy crises such as sanctions to boost their approval among citizens (Chatagnier, 2012). Sanctions are prime opportunities for hawkish foreign policy practitioners to advocate for increased military spending (Whitten and Williams, 2011) and justify diversionary tactics abroad (Jung, 2024). On top of these, spending on the military may also have a (short-term) positive economic impact, countering the economic shocks by increasing demand for goods and services and boosting employment particularly in the defense sector (Mintz and Stevenson, 1995; Whitten and Williams, 2011; cf. Yesilyurt and Yesilyurt, 2019).

Zimbabwe’s reaction to Western sanctions in the early 2000s illustrates this mechanism. Facing sanctions, the Mugabe government called on the army to ‘speed up’ land reform and to create ‘a general mood and psychology of obedience to law and order’ (McGreal, 2001). Jealousy Mawarire, a former close ally of Mugabe, later acknowledged that the land reform was spearheaded by the military with the primary goal of creating what they called ‘liberated zones’ in case the opposition party won the next elections. Mawarire claimed that ‘[t]he people who took the farms were not farmers, but [. . .] were either war vets or security services officials who were under instruction to KEEP the land’ (Pindula, 2021). Aside from deploying and compensating the military, the Mugabe government, facing Western arms embargoes and believing the West was aiming at regime change, also emphasized the need to step up military training and procure new weapons. The then-defense minister argued that ‘[m]ilitary preparedness should always be top priority, even during peacetime. While there may be no direct military threats to Zimbabwe today, there may be one tomorrow’ (as cited in Tambudzai, 2011). In the mid-2000s, the Ministry of Defense signed major contracts with several Chinese defense companies (Tambudzai, 2011). As a result, in the aftermath of sanctions, the relative military spending in Zimbabwe rose from 2.5% in 1999 to 3.4% in 2000, 2.9% in 2001, and 7.1% in 2002 (SIPRI, 2019).

### Rise in relative military spending that dissipates over time

Taken together, both the signaling and power-consolidation rationale suggest that target states have incentives to increase their relative military spending. For several reasons, the increase should only be short-term and dissipate over time. First, the benefits and incentives to signal resolve should cluster in the early stage of sanctions since, as a crisis prolongs, the issues of uncertainty and informational asymmetry tend to be alleviated (Reiter, 2009). Second, once target states endure the initial economic shocks, either the economy returns to normal (e.g. sanctions are lifted) or it adjusts to the new environment accordingly (e.g. third-party states bust the sanctions). As such, the economic impact of sanctions ‘should fade out as sanction-busting states and black market participants exploit opportunities for arbitrage and thereby mitigate some of the damage caused by sanctions’ (Gutmann et al., 2023: 1216). Relatedly, after an increase in relative military spending, the need to further boost the military would weaken and be dominated by other priorities. Finally, since the shocks to leaders’ political power are concentrated in the early stage of sanctions (Dizaji and Farzanegan, 2021; Peksen and Drury, 2010), the incentive to increase relative spending to consolidate power also weakens over time.

One potential counterargument is that the short-term rise in relative military spending could be attributed solely to the economic shocks a target state encounters during the early years of economic sanctions. We do not dispute the negative economic consequences of sanctions (and also show them empirically later in the article). Our theoretical expectation, however, is that a state that encounters a sanction shock tries to maintain or increase *absolute* military spending or cut it at a slower pace than the (negative) growth rate of its economy, thereby resulting in the increase of *relative* military spending. It is the trading of butter for guns rather than the other way around in the face of economic pressures that is the focus of our argument.

Another counterargument could be that sanctions push up the prices of commodities the military needs, at least in the short run. Even if military commodity prices rise, however, we still need to explain why states are willing to take the distorted prices. After all, if sanctioned states expect the prices to drop after some time (or even to return to normal), they could adopt the policy option of waiting out. Our argument suggests that the signaling and power-consolidation incentives make this option unattractive. Therefore, we arrive at the following two hypotheses:

*Hypothesis 1:* All else being equal, states that experience sanction shocks increase their military expenditure as a share of the economy.
*Hypothesis 2:* The impact of sanction shocks on relative military expenditure dissipates over time.

## Research design

To test the two hypotheses, we apply dynamic panel modeling to account for the autoregressive process of military spending (more details later). In the main models, we use Autoregressive Distributed Lag (ADL), which is particularly useful for investigating the impact of sanctions over time. In robustness checks, we apply alternative methods including impulse response function and generalized method of moments estimation.

### Relative military spending

The key outcome variable is military expenditure as a share of GDP. These data come from the SIPRI Military Expenditure Database (SIPRI, 2019) that covers states’ relative military spending from 1949 to 2018. We also report in the Online Appendix results using military spending as a share of government expenditure as the dependent variable, though the data cover a much shorter period (1988–2018). While the results are substantially similar, we use the share of GDP as our main outcome variable because states often have the fiscal option of increasing borrowing (Oatley, 2015; Shea, 2016).

**Figure 1.**
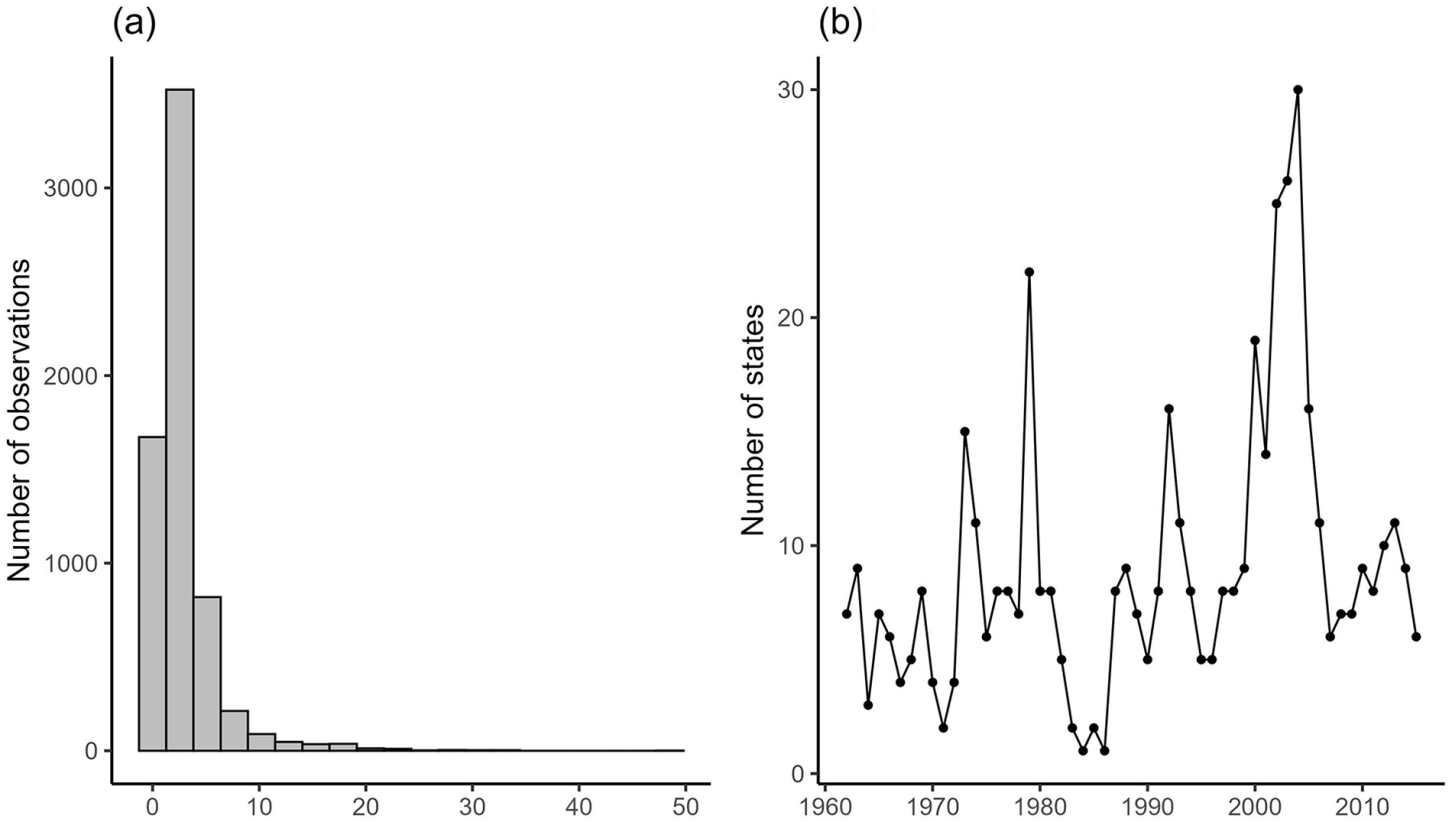
Distributions of the dependent variable and primary explanatory variable. (a) The left panel shows the histogram of relative military spending (% of GDP). (b) The right panel shows the number of states encountering sanction shocks over time.

We use the SIPRI data given it covers a long period of time for most countries across the world. In particular, it offers comprehensive estimates for countries such as China and Russia where official military spending data are not necessarily available or reliable.^3^ The distribution of the military expenditure data is shown in Figure 1, panel (a). The plot suggests that most states spend less than 10% of their GDP on the military, though it is also highly skewed to the right.^4^ There are 203 observations (around 3.5% of the data) that have military expenditure as a share of GDP greater than 10%.^5^

Importantly, the relative military spending data appear to follow an autoregressive process. While more detailed discussions are left to the Online Appendix, two points should be noted here. First, the results of the panel bootstrap unit test suggest that the null hypothesis that all series have a unit root cannot be rejected (p-value > 0.17). Second, given the test results, we take the first-order difference of the relative military spending data and plot the auto-correlation function (ACF) and partial auto-correlation function (PACF). The plots show that the auto-correlation is not statistically significant after the first lag.^6^ Taken together, the results suggest that we should model the military spending data at least as an order 1 autoregressive process.

### Sanction shocks

To identify sanction shocks, we use the Threat and Imposition of Economic Sanctions (TIES) Data (1945–2005) by Morgan et al. (2014) and the EUSANCT Dataset (1989-2015) by Weber and Schneider (2020).^7^ Our operationalization of the *Sanction shocks* variable takes into account three dimensions, namely heterogeneity in issue salience, the (expected) costs of sanctions, and the varying effects over time. First, not all sanctions are equal. Sanctions concerning high-salience issues are more likely to generate (perceptions of) threats and shocks (Ang and Peksen, 2007). These sanctions are more likely to be enforced more harshly (Ang and Peksen, 2007; Early and Preble, 2020). As such, target states should expect the shocks to be more concrete and persistent for high-salience issues. To account for this, we follow Peterson (2021), who argues that trade disputes and other ‘low politics’ issues are less likely to cause concerns about security and domestic challenges to leaders’ power. We, therefore, use the issue variable and exclude sanctions where the issue is purely one of, or a combination of, the following types: releasing citizens, property, or material, deterring or punishing drug trafficking practices, improving environmental policies, changing trade practices, and implementing economic reform.

Second, we account for sanctions’ (expected) economic and political costs. For economic costs, we use the (anticipated) target economic costs variable in TIES, which identifies costs into three categories: minor, major, and severe.^8^ We code the last two categories as high economic costs. For political costs, we code them as high when the sanctions were threatened or imposed multilaterally. This is because existing studies have shown that multilateral sanctions entail a more substantial destabilizing effect on target regimes (Allen, 2008b; Grauvogel et al., 2017). Using these two variables, we only consider sanctions where either the economic costs or the political costs are high.

Finally, there is a time dimension since sanction shocks cannot last forever. We carefully differentiate three scenarios: sanction threats, sanction impositions that lasted less than one year, and sanction impositions that lasted more than one year. In the first two scenarios, we code the first year as sanction shocks since we should expect sanctions that were only threatened but not imposed or were lifted within one year to have limited impact. In the last scenario, we code the first two years as sanction shocks. We choose the two-year benchmark since studies show that it is a critical time to witness sanction success (Dizaji and van Bergeijk, 2013; Peksen and Drury, 2010). It is, therefore, a reasonable period to expect substantial economic or political shocks to target countries.^9^ This way, our coding rule can separate sanctions that lasted longer from those of limited impact or shorter duration. For example, our method identifies China in 1979 and 1989–1990 as experiencing sanction shocks since, in the former case, the Soviet Union threatened to sanction China but then backed away from it in late February.

We plot the number of states that are coded as encountering sanction shocks over time in Figure 1, panel (b). On average, our coding scheme identifies around nine countries each year as encountering sanction shocks. The sharp rise from 2000 to 2003 in the figure is driven by the expansion of states that encountered new or harsher sanctions (e.g. Venezuela and Zimbabwe in 2000). It should also be noted that for scholars who are concerned that merging TIES and EUSANCT creates discrepancies, we have carefully accounted for their different coding rules, as mentioned earlier. Indeed, the figure showcases that in the years 2005 and 2006, there is no substantial change in the number, which provides at least some prima facie evidence that our coding scheme works as planned.

Once we have coded the *Sanction shocks* variable, we further code an additional variable named as *Sanction without shocks* to indicate a state that is under economic sanctions in a given year but is not encountering sanction shocks.

### Control variables

To alleviate the concerns of spurious correlation, we control for a number of variables. First, rising tensions are positively associated with both economic sanctions (Drezner, 1998) and higher relative military spending. We thus control for whether the target states are involved in any interstate or intrastate conflict. To code whether a state is involved in a conflict, we use the UCDP/PRIO Armed Conflict Dataset (1946–2015) (Gleditsch et al., 2002). We do not differentiate types of conflict and code this variable as binary since we do not have theoretical expectations about how their effects would vary. In the robustness checks, we also rerun the analysis using the number of conflicts a state is involved in.

Second, a state’s existing level of economic integration can condition how severely they are affected by the sanction shocks and how they choose to adjust the level of military spending. This is because more integrated states are able to discount the costs of economic disruption more heavily (Kinne, 2012; Peterson, 2020; Zeng, 2020). Moreover, a country’s economic integration can strongly affect their expectation about current and future abilities to produce or purchase weapons and related strategic materials (Dorussen, 2006; Goenner, 2010). We therefore control for a state’s level of economic integration in the global economy using the measurement proposed in Zeng (2024).

Third, we control for other resource-related factors. The number of inhabitants of a country gives us a handle on the size of the country, which may proxy for a state’s ability to endure economic disruption. We also control for the amount of aid a state receives relying on net official development assistance and official aid (1960–2019) data from World Bank (2022). We take the logarithm of the population and foreign aid data.

Fourth, we use the Autocratic Regime Data (1946–2010) by Geddes et al. (2014) to code whether a country is a democracy or not. This is because the political shocks of sanctions could be heavier for democracies (Allen, 2008a). Meanwhile, democratic governments may face more constraints when considering channeling more money to the military (Fordham and Walker, 2005). Finally, we control for timing by including either year fixed effects (most models) or by distinguishing between the Cold War and Post-Cold War eras by coding whether the year is before 1991. The summary statistics for all variables are presented in Table 1. We can see that states encountering sanction shocks tend to spend proportionally more on the military. They are also more integrated in global trade networks and more likely to be involved in conflict.

**Table 1.** Summary statistics: 1962–2015.

Variables	Sanction shocks		p-value^a^
	FALSE N = 5,662	TRUE N = 362	
*Relative military spending*, mean (SD)	2.74 (3.24)	3.62 (3.81)	<0.001
*Absolute military spending*, mean (SD)	7.00 (2.24)	7.59 (2.39)	<0.001
*GDP*, mean (SD)	17.87 (1.89)	18.22 (1.89)	0.003
*Conflict*, n (%)			<0.001
FALSE	4,041 (71%)	214 (59%)	
TRUE	1,621 (29%)	148 (41%)	
*Integration*, mean (SD)	1.12 (0.28)	1.16 (0.22)	0.019
Log *Population*, mean (SD)	9.14 (1.55)	9.75 (1.58)	<0.001
Log *Aid*, mean (SD)	4.03 (2.85)	4.66 (2.76)	<0.001
*Democracy*, n (%)			0.9
FALSE	3,419 (60%)	217 (60%)	
TRUE	2,243 (40%)	145 (40%)	

a Wilcoxon rank sum test; Pearson’s Chi-squared test.

## Results

With the foregoing data in hand, we specify the full ADL model as follows:

$$\begin{array}{c} Relative\;military\;spending=\phi_{0}+\underset{m=1}{\overset{n}{\sum }}\alpha_{m}L^{m}(Relative\;military\;spending)\\ +\underset{k=0}{\overset{p}{\sum }}(\beta_{k}L^{k}\left(Sanction\;shocks\right)+\gamma_{k}L^{k}\left(Sanction\;w/o\;shocks\right))\\ +\underset{i=0}{\overset{r}{\sum }}\delta_{i}L^{i}\left(Control\;variables\right)+\epsilon \end{array}$$

where $L^{k}$ denotes lagging a variable by k years. There can be multiple lags for the key variables of interest as well as the control variables. We search through all combinations of lags (up to lagging by three years) for each independent variable using the ADL model and present the one with the lowest Akaike Information Criterion (AIC) score in Table 2.^10^

**Table 2.** Regression results with 95% confidence intervals: 1962–2015.

	Dependent variable:		
	Relative military spending (1)	Absolute military spending (2)	GDP (3)
Lag (*Relative military spending*)	0.675** (0.656, 0.695)		
Lag (*Absolute military spending*)		0.881** (0.870, 0.892)	
Lag *GDP*			0.948** (0.942, 0.954)
*Sanction shocks*	0.234* (0.033, 0.435)	0.031* (0.004, 0.058)	−0.011* (−0.020, −0.002)
*Sanction w/o shocks*	0.142* (0.023, 0.261)	0.016* (0.0004, 0.032)	−0.001 (−0.006, 0.005)
*Conflict*	0.434** (0.313, 0.555)	0.033** (0.017, 0.049)	−0.021** (−0.027, −0.016)
*Integration*	−0.024 (−0.503, 0.456)	0.108** (0.043, 0.173)	0.071** (0.049, 0.094)
Log *Population*	−0.359* (−0.657, −0.061)	0.035^†^ (−0.005, 0.076)	0.015* (0.003, 0.027)
Log *Aid*	0.036* (0.006, 0.065)	−0.001 (−0.005, 0.003)	0.001* (0.00001, 0.003)
*Democracy*	0.061 (−0.089, 0.212)	0.008 (−0.012, 0.029)	−0.004 (−0.011, 0.003)
Fixed effects	Two-way	Two-way	Two-way
Observations	5,971	5,851	7,738
R^2^	0.466	0.824	0.935

† *p* < 0.1. *p < 0.05. **p < 0.01.

### Main results

In Table 2, we present results of the main models. In Model 1, we regress military spending as a share of GDP on the predictors and country and year fixed effects. We find a positive and statistically significant effect for the *Sanction shocks* variable. This result suggests that in the short run, states encountering sanction shocks tend to increase their relative military spending, providing strong support for Hypothesis 1. Directly interpreting the results from the table is complicated by the fact that a given year’s impact gets carried over into the next year via the lagged outcome variable. To better illustrate the impact over time, we use simulation methods to estimate the marginal effects of the sanction shocks. We begin by drawing 1000 samples of coefficient estimates from the results of Model 1. Holding other variables at their median values, we can calculate the expected values of relative military spending for the cases when states encounter or do not encounter sanction shocks. Importantly, since the lagged outcome variable is included on the right-hand side of the equation, we iteratively update its values over time when estimating the marginal effects for each year. This way, we can estimate the expected values of the different cases over several years. Taking the difference between the two sets of values (e.g. states encounter vs. do not encounter sanction shocks), we obtain the marginal effects of sanction shocks over time.

**Figure 2.**
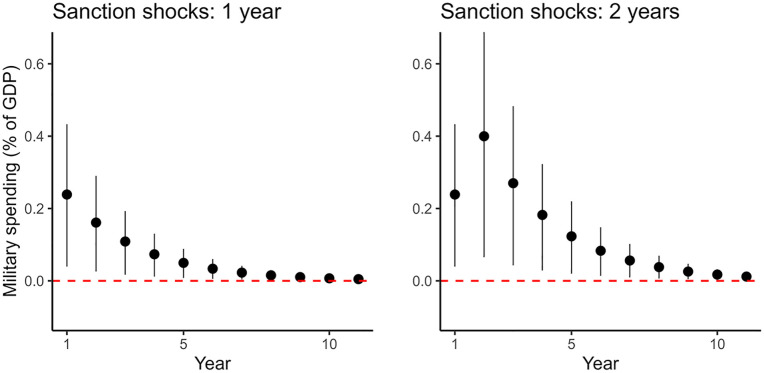
Marginal effects of sanction shocks with 95% confidence interval. The y axis denotes changes in the expected value of military spending when a country experiences new sanction shocks. The left and right panels show the effects for countries encountering sanction shocks for 1 year and 2 years respectively.

Applying this method, we simulate the marginal effects for two scenarios: states that encounter sanction shocks for one or for two years. The results are shown in Figure 2. For illustration purposes, we plot the marginal effects for the first 11 years. For states that encounter sanction shocks, their relative military spending increases by around 0.24 percentage points in the first year, which is substantial given that the average military expenditure of states facing no sanctions is 2.7% of GDP. Compared to this baseline expenditure, sanction-shocked states thus boost their military spending by around 9% in the first year.

If the sanctions are lifted in the second year (the left panel), then the marginal effects start to decline in that year. Starting the seventh year, the average effect gets very close to 0 (less than 0.023%, one tenth of the first year’s effect). If a sanction shock persists into the second year (the right panel), then the estimated marginal effect increases in the second year to 0.4% and only declines from the third year onward.

Taken together, these results strongly support our two hypotheses: we see a short-term increase in relative military spending in response to sanction shocks (Hypothesis 1) that dissipates over time (Hypothesis 2).

### Additional evidence and robustness checks

The increase in relative military spending that we have shown can result from both an increase in absolute military spending and a decrease in GDP. As mentioned previously, assuming sanction shocks lead to economic decline, there are two possible reasons for us to observe the increase in relative military spending: (a) absolute military spending remains unchanged (or even increases) and (b) absolute military spending reduces, but at a slower rate than the decline of the economy. To analyze to which extent these two mechanisms are at play, we rerun the analysis using military spending in absolute terms and GDP as dependent variables. We use the values of both variables adjusted by purchasing power parities (PPP).^11^

Models 2 and 3 in Table 2 present the results. In line with the existing studies on sanctions’ economic impact (Neuenkirch and Neumeier, 2015), we find strong evidence that sanction shocks reduce GDP (Model 3). Meanwhile, in Model 2, the coefficient for sanction shocks remains positive and statistically significant when using absolute military spending as the dependent variable. We, therefore, find evidence of mechanism (a). That is, the decline in relative military spending is a combination of an increase in absolute military spending and a decline in GDP.

Moving on to robustness checks, we rerun the analysis by: (a) using the number of conflicts a state is involved in instead of the binary measure; (b) using military expenditures as a share of total government expenditure; (c) using polity IV data to measure democracy; (d) using country fixed effects with a Cold War dummy; (e) using robust standard errors to account for heteroskedasticity and serial correlation; and (f) using only the TIES dataset for measuring sanction shocks; (g) excluding potential outliers (relative military expenditure greater than 10%); (h) using 5 years instead of 2 years to measure sanction shocks. As shown in the Online Appendix (sections F to M), the results remain substantially similar to those of the main models across all these robustness checks.

We also apply two different estimation methods: impulse response function and Generalized Method of Moments (GMM) estimation. Impulse response functions are used to estimate how a dynamic system reacts to an impulse, in this case, a sanction shock. Because the military expenditure data are non-stationary (see Section A of the Online Appendix), we use the first differences of the dependent variables for this approach. The GMM model allows us to address possible concerns over Nickell’s (1981) bias, i.e. dynamic models with individual fixed effects may result in downward-biased estimates. Both approaches confirm the central finding that sanction shocks lead to higher relative military spending.

## Conclusion

We have argued that sanction shocks increase target states’ *relative* military spending in the short run, with this effect dissipating over time. This is because by channeling proportionally more resources into the military, leaders can boost their bargaining leverage against foreign adversaries and secure their tenure by improving their ability to co-opt and repress. As these incentives weaken over time, the effect of sanction shocks on relative military spending should dissipate over time. Applying dynamic panel modeling, we have found strong support for both hypotheses.

The results can help advance studies on sanctions and military spending. Whereas existing research demonstrates that sanctions reduce states’ military spending in absolute terms, we find contrary evidence suggesting that when adjusting for PPP, the effect is positive and statistically significant. More importantly, so far, we have not known whether states spend proportionally more or less on the military. This article furthers the field by showing that sanctioned states – especially in the first years after sanctions – spend comparatively more on the military despite a smaller budget. This is direct evidence that in the short run many sanctioned states decide the trade-off between guns and butter in favor of the former. Given that the initial stage of sanctions is critically important for their success, this result can help explain why some sanctions are not as effective or destabilizing as expected.

Illustratively, our results may help explain Russia’s reactions to the unprecedented sanctions imposed against it in 2022. Initially, Russia planned to cut its defense expenditure from 4.679 trillion rubles in 2022 to 3.473 trillion in 2023. However, as Western sanctions took a further bite on the economy, Putin reversed this planned cut and instead chose to increase the spending by over 43% in the following year (Bloomberg News, 2022). For 2025, Russia’s military spending as a share of GDP is expected to rise to 8% (Korsunskaya and Bryanski, 2024). While to a large extent, this increase is made necessary by the war it wages against Ukraine (a factor we control for in our analysis), the case still illustrates our logic. Russia manages to shift resources to the military in the face of heavy sanctions; it signals resolve and unwillingness to back down, which likely would cause high domestic costs for Russia’s current political leadership.

That said, there are several directions that future research could pursue. For one, studies could explore the factors that moderate the relationship between sanction shocks and (relative) military spending. Regime type is such a potential moderator. Democracies may be more responsive to the welfare of the general public and face more institutional constraints when reallocating resources than autocracies (Ali and Solarin, 2020; Cappella Zielinski and Schilde, 2019; Hewitt, 1992). Existing studies have also shown that autocratic leaders (personalist ones in particular) tend to focus on controlling and buying off the military to insulate themselves from coups and deter potential challengers (Acemoglu et al., 2010; Bove and Nistico, 2014; Escribà-Folch, 2012). Relatedly, it is also possible that the impact of sanction shocks could be contingent upon states’ involvement in international or civil conflict, which has also been shown in our analysis as having a positive and significant impact. Building on these results, future studies can theorize and test how the impact of sanction shocks varies across different institutional arrangements and is dependent on conflict involvement.

Moreover, scholars can explore the indirect mechanism of sanction shocks. For instance, it is possible that sanctions first shock states’ economic integration in the global market. This can then affect their options in terms of finding alternative markets and suppliers for weapon production, thereby rearranging their incentives to ratchet up or cut back military spending. Scholars can examine how substantial the sanction shocks on economic integration can be and then apply methods such as mediation analysis (Imai et al., 2010) to test the mechanism. Pursuing this line of research in these or other directions would be important to shed additional light on sanctions as a frequently used foreign policy tool.

## Notes

1. Additionally, sanctions can exacerbate exchange rate volatility, which poses additional challenges for cross-country comparison of absolute military spending patterns (Batmanghelidj, 2022; Kofman and Connolly, 2019).

2. Iraqi military spending relative to GDP was well over 100% in the first three years (1991–1993) after embargo imposition (US Department of State, 2000). The absolute values are 1.77, 1.8, and 1.85 billion US dollars respectively. Even if we use the more conservative estimates of 1.4 billion per year (Lopez and Cortright, 2004: 98), the ratios were still above 100% in these three years.

3. It also offers estimates for countries such as Israel and the United Arab Emirates. For details of the estimation methods, see SIPRI (n.d.). It should also be noted that missing data is still a prevalent issue for some countries, particularly for sanctioned countries that are more likely to have high military spending (Cuba, North Korea, and Iraq in the 1980s and 1990s).

4. There is one observation with military expenditure as share of GDP greater than 1 in the SIPRI data: Kuwait in 1991 (the value is 1.17). There are 89 observations with military expenditure equal to 0: Costa Rica (1949–2018) and Panama (2000–2018). For illustration purposes, we do not include the Kuwait 1991 case in the histogram. The Online Appendix offers additional plots comparing countries over time.

5. The Online Appendix provides a list of all 203 country-years. Out of these 203 observations, 23 (around 11.3%) are cases where a country experiences sanction shocks.

6. Relatedly, we rerun the panel unit root test on the first-differenced series and find very strong evidence to reject the null (p-value < 0.01).

7. We use TIES for the period between 1945 and 2005 and EUSANCT for the period between 2006 and 2015. As discussed later in the article, we use the issue and economic cost variables from TIES. The exact categories for these two variables in EUSANCT are different. We have carefully harmonized them as much as possible. In the robustness check, we also rerun the analysis using only the TIES data and the results are substantially similar.

8. In TIES, there are two variables for economic costs. *Anticipated target economic costs* refer to the costs of sanction threats, while *Target economic costs* refer to the costs of sanction impositions. We have matched them carefully. There are two additional complications with using the *Target economic costs* variable. First, there are many missing values for these variables in TIES. We try to alleviate this problem by selecting only the maximum costs for each country year. We believe this is a reasonable choice for teasing out cases with substantial costs since they are more likely to be reported. Second, EUSANCT uses a different category to code target costs. Specifically, it codes the costs into six categories. We code the first two as minor costs, the next two as major costs, and the last two as severe costs.

9. See the Online Appendix for additional robustness checks using a five-year benchmark. Our choice of using a two-year benchmark in the main model also aligns with other studies on security shocks and external threat perception. For instance, Oatley (2015: 44) codes security shocks as ‘two-year events from the date they occur’.

10. Given our theoretical expectation and the autoregressive nature of the military spending data, we specify that there is at least one lag for the outcome variable (as shown in the formula). Using Bayesian Information Criterion (BIC), this model is still the best.

11. We adjust absolute military spending by PPP since GDP in PPP terms aligns better with the existing literature on sanctions’ economic impact. We also keep the same specifications as in Model 1 to facilitate comparison. That said, to fully test the respective effects, both models would need to be adjusted. For instance, in Model 2, one should consider accounting for GDP. The main results for the *Sanction shocks* variable, however, remain the same.
